# What is socially constructed about race? A cross-national study of racial perception and categorization

**DOI:** 10.1371/journal.pone.0351831

**Published:** 2026-08-12

**Authors:** Sayaka Osanami Törngren, Marcus Nyström, Emi Moriuchi

**Affiliations:** 1 Malmö Institute for Studies of Migration, Diversity and Welfare, Global Political Studies, Malmö University, Malmö, Sweden; 2 Lund University Humanities Lab, Lund University, Lund, Sweden; 3 E. Philip Saunders College of Business, Rochester Institute of Technology, Rochester, New York, United States of America; National Institutes of Health, University of the Philippines Manila / De La Salle University, PHILIPPINES

## Abstract

This study investigates the social construction of race by examining how racial categories are understood and applied by college students in Japan, Sweden, and the United States. Addressing a gap in empirical research on racial appraisal versus self-identification, the study employs a comparative and contextual approach using three types of data: open-ended definitions of race, racial and skin-color ascription tasks, and eye-tracking measures. The findings reveal both cross-contextual similarities and differences in how race is conceptualized and enacted. While respondents across all sites commonly defined race through physical characteristics and imposed categorization, contextual nuances etourismmerged—particularly in the use of cultural and ethnic framings. The study also demonstrates that the same faces were racially ascribed differently across settings, underscoring the fluidity of racial perception. Although eye-tracking data did not show variation in visual attention to facial features, the overall results provide compelling evidence for the contextual flexibility of racial categorization. These findings contribute to a deeper understanding of race as a socially constructed phenomenon and highlight the need for further comparative research on racial perception and identity across diverse sociohistorical contexts.

## Introduction

Despite the progresses made in the past decades in raising awareness towards racism, race and racial identities, which “erroneously essentializes characteristics (including phenotypical characteristics), creating prescribed (but changing) social categories that individuals may or may not self-identify with and that others may or may not “accurately” assign” [1], continue to matter in the global context more than ever. The murder of George Floyd by Derek Chauvin a White police officer on May 25, 2020, in the US inspired a surge of Black Lives Matter (BLM) demonstrations around the globe. The Black Lives Matter (BLM) movement, which began in the United States after Alicia Garza’s statement “Our lives matter,” and was co-founded by Patrisse Cullors and Opal Tometi in response to the 2012 murder of 17-year-old African American Trayvon Martin by George Zimmerman, gained unprecedented global attention and support during 2020 [2–4]. The same year, 2020, the Covid-19 outbreak around the globe brought attention and awareness to anti-Asian racism on a global scale [5]. These incidents suggests that people’s lived experiences, particularly Black and Asian individuals (e.g., BLM, #stopAsianhate), are more susceptible to racist incidents. This susceptibility could be due to globalization and the ubiquitous presence of social media. However, these negative experiences do not happen in one particular country but are, rather, a worldwide phenomenon that happens across different contexts [6].

Today we have a strong consensus that race is a social construct and an analytical concept that is multilayered and multifaceted [1,7]. Recent global events have amplified discussions about racism and underscored the need to understand how race is conceptualized, and how racial groups are formed and perceived across different societies, shaping racial hierarchies and inequalities in diverse ways. There have been calls for a comparative understanding of race [6,8,9] and comparative analyses of how race as a category functions in different contexts are emerging [10,11], yet little is known about how particular understandings of racial categories and practices of division in one context relate to or differ from the understandings of racial categories in another. This article is our first attempt to address this knowledge gap.

This paper is first of its kind to empirically examine what is socially constructed about race through examining how the concept of race is understood and how flexible racial categories are across three distinct contexts – Japan, Sweden and the US. Scholars have repeatedly emphasized that individuals are socialized to perceive race—including their own and others’—in ways that vary across contexts. These variations are shaped by broader societal norms, as well as by historical legacies and contemporary conditions—including colonial histories, national racial projects, and ongoing structural inequalities—that provide the backdrop against which people racially identify and are classified. [12–16].

While Sweden operates under a color-blind myth in which the word *race* is avoided even though discrimination and inequality still manifest along lines of social categorization [17,18], the US is a society where race and racial awareness are salient, especially in terms of the Black and White divide [19]. Japan, on the other hand, has a unique way of engaging with white supremacy, placing people socially and racially categorized as ‘White’ or ‘Japanese’ at the top of a racial hierarchy [20]. Grounded in the conceptualization of race as a socially constructed category, this study hypothesizes that individuals’ understandings of race—as well as the ways in which they identify racially and classify others—are shaped by socialization processes within specific sociocultural contexts. To investigate this, we asked a sample of Japanese, Swedish and American college students (N = 88): (1) open-ended responses in which participants define the concept of race, offering insight into their underlying conceptual frameworks; (2) racial ascription tasks in which participants assign racial categories to facial images [21], together with skin color, using the NIS (New Immigrant Survey) skin-color scale [22] revealing patterns of classification and perception; and (3) eye-tracking data that captures visual attention during these tasks, providing a behavioral lens on how racial cues are cognitively processed. Together, these data enable a multidimensional exploration of how race is socially constructed, interpreted, and enacted across different settings.

There are very few empirical studies which examine how racial appraisal differs from self-identification [23]. This empirical inquiry into the processes of racial categorization offers the promise of a unique contribution to comparative and contextual understandings highlighting race as a social construct and contributes to our understanding of the unwritten social patterns that inform racial attributions in practice [24].

## Race and racial categories

In this article we define race as one of the modes of categorization – a process which reflects a mechanism of structural hierarchies based on phenotype [25]. When we see a face, we process what the face symbolizes and therefore it is seemingly improbable to be racially color-blind [26]. Research in social psychology has indeed provided conclusive evidence on face processing and how the presentation of a face can evoke social-category information, related schemas and stereotypes [27–29].

Loveman [30] suggests separating our understanding of race as a category and race as a group, since belonging to one racial category is not always equal to identifying and belonging to the racial group [31]. Loveman argues for the need to understand race as a category of practice and to build a framework that focuses attention on “the processes of boundary construction, maintenance, and decline – a comparative sociology of group-making” [30]. The categorization of individuals relies on visual cues [32]; how we are ascribed certain racial groups depends on the context. This is because stereotypical expectations and beliefs about who falls into the category are contextual. Who counts as having a minority or a majority status is based on visibility shifts [33] because which phenotypes are deemed important and are considered visible depends on the contextual and stereotypical knowledge. In other words, salience of the visibility in the context is crucial in understanding who is categorized as what. In this study, cultural schemas are defined as “mental constructs” and function as representations and processors of information that are central in understanding what expectations and beliefs are attached to the categories. Schemas reveal how we see and understand race – the cognitive perspective on how we put people into different racial categories [16,34]. Race is not an inherent trait of individuals. Since race is a social construct and racial perceptions influence structural hierarchies, national politics determine which racial categories are recognized and deemed significant within a country [35]. Therefore the classification and ascription of racial categories are highly dependent on the specific time and place, even within the same country [12,18].

## Challenges of comparative analysis of race

Measuring race, analyzing racial boundaries and predicting their shaping and reshaping are methodologically challenging [36,37]. Vargas and Kingsbury [24] argue that to develop a broad understanding of how race operates to shape people’s life chances in society, we should examine not only individuals’ personal racial identities in survey research but also whether such identities are commonly either confirmed or contested by others. Indeed, racial categorizations matter differently in North America, Asia, the Middle East and Africa – which groups are racialized and how race affects individual life chances both negatively and positively because of the structure of power, privilege and oppression attached to the visible cues [38]. How individuals are socialized to see race and perceive their own and others’ races are different across the three contexts of this study. Since race is today understood as a social construct, the historical and the contemporary conditions and contexts affect how people racially identify or are racially classified [12–16].

Moreover, the contexts of immigration and ethnic and racial diversity in the three countries that we are comparing in this article are significantly different in Japan and Sweden compared to the US. The latter is often described as a country built on immigration, while Japan and Sweden have lived under the myth of homogeneity despite the presence of an indigenous population. Sweden has become a country of immigration, with ethnic and racial diversity, especially since the 1980s when asylum-seekers from non-European countries became the dominant category of immigrants. Even though immigration to Japan has increased over recent decades and is expected to continue increasing, the country’s immigration policies are restrictive. As immigrants are predominantly from other Asian countries and/or of ethnic Japanese origin, the ethnic and racial diversity of the country is not always as visibly prevalent as it is in the US or Sweden. In 2022, 14% of the total population in the US, 20% in Sweden and 2% in Japan are foreign-born. The largest number of foreign-born people in the US have their origins in Mexico, India and China; in Sweden they are from Syria, Iraq and Finland and, in Japan, from China, Korea and Vietnam [39].

Since perceptions of others are always relational, these differences affect our awareness of diversity and the boundaries of ethnic and racial groups [31,40,41]. While Sweden is still operating under a color-blind myth and is avoiding addressing race as one of the mechanisms of inequality [17], the US is a society where, despite the persistence of racial inequality, race and racial awareness are salient, especially in terms of the Black and White divide [19]. On the other hand, Japan has its unique way of looking at race in terms of Whiteness and Japaneseness [42]. In the US, the history of segregation still affects the Black community while, in Japan, the history of the colonization of China and the Korean peninsula still forms an understanding of the superior Japanese and the inferior “Asians” [42,43]. These historical and contextual differences in race relations across the U.S., Sweden, and Japan clearly shape how participants conceptualize race, influencing the salience of racial categories, the perceived boundaries of groups, and the meanings attached to racial identity in each setting.

The use of the word “race” is normalized in the US, where respondents are constantly exposed to the administrative practices of reporting their racial identity [10,35]. However, reporting and talking about race is not self-evident in the Japanese and Swedish contexts, where administrative data and the practice of self-identification do not exist. Thus, formulating the word race, due to the different socio-historical contexts, is one of the difficulties of engaging in transatlantic research on race.

Moreover, the way we define race academically, is not the same as how the word “race” is understood or used in different contexts. Morning and Maneri’s study [10] comparing Italy and the US highlights how the term race itself may not mean the same thing or be used in the same way. In order to understand race as a mode of categorization, our study therefore seeks to decipher the meaning of the word race in different contexts, together with how visible genetic features are categorized (skin color and other visible phenotypes).

In formulating the survey in three languages, we consulted with our collaborators in order to ensure that the wording captured a similar understanding of the term across the three contexts. We chose *jinshu* in Japanese as this is the most commonly used word, historically. There is a consensus that this word indicates the categorization of people according to phenotypes and the systems of subjugation that Japan was both the victim and the perpetuator of during and after WWII [44,45]. We chose the translation *rasifiering* in Swedish. The Swedish Government erased all the mentions of the word *ras* from the legislation and the word *rasifiering* (racialization) and *rasifierad* (racialized) are today commonly used to replace the word “race” [17,46,47]. We chose these translations because, in the contexts of Japan and Sweden, they are understood to refer to socially constructed groups that experience racism, discrimination, or are otherwise identified as minoritized. In this study, we cannot validate whether the terms are perceived as equivalent across languages; therefore, we focus on respondents’ own understandings and definitions of race, jinsū, and rasifiering to clarify what these terms signify within each specific context and to compare how different faces are racially ascribed across contexts.

Below, we further discuss how these choices of translating the words show a common understanding of race across contexts in the analysis.

## Cultural influence and racial bias on eye movements

Human beings tend to fixate on each other’s eyes, since our attention to eyes is innate and because eyes play a crucial role in impression formation [48]. However, how much visual attention we pay to the eyes and other parts of the face may depend on what is culturally and socially determined and categorized as visibly important in a context, due to the specific socialization and cultural practices [33,49,50]. Research shows that own-race faces are recognized more accurately than other-race faces, a finding known as the “own-race bias” [51,52]. Own-race bias and cross-race effects are explained through a lack of contact with other races or through differences in the cognitive process of recognizing own- and other-race faces [50,53]. Several studies have explored eye movements and the own-race advantage in face recognition [54–57].

Previous eye-tracking studies have shown distinct patterns of eye movement for people raised in different geographical locations and/or in different cultures [read 50 for an overview, 58, 59]. However, there are also cross-cultural studies which found that there are no visual preferences and different eye-movement patterns such as location of first fixation, distribution of fixations or dwell time on different parts of the face [e.g., 60–62]. There is also evidence for differentiated and preferential processing of own- and other-race faces in research within a single culture and context. These studies indicate greater visual attention to the eyes of a person’s own group compared to other groups [54,63–65].

## Method

### Data

The data analyzed in this study are part of an experimental eye-tracking data collection on the perception of and attitudes towards tourism photographs and faces; this is a sub-project of a larger project which explores the role of tourism in multicultural societies in acting for the inclusion and representation of diversity. The project was funded by the Swedish Research Council for Sustainable Development (Svevnska Forskningsrådet), FORMAS, grant number FR2018/0010. The study was approved by the Swedish Ethics Authority (Dnr 2019–05597) and the RIT Institutional Review Board (HSRO #03102919), and was waived in Japan. The data inquiry consisted of two parts; the first part showing images of different places and faces and the second part being a survey containing background and attitudinal questions on how the participants understand concepts of race and ethnicity and view diversity and multiculturalism. Below we provide a detailed account of the data-collection procedures and the parts of the data which we analyze in this article.

Participants were welcomed and asked to read information about the experiment and sign a consent form. Written informed consent was obtained from all participants. The consent form explained that the study is part of a project aimed at understanding perception of and attitudes towards tourism photographs and faces. Participants were taken to the experimenter room and given a brief introduction to the experiment. We used identical equipment in our data collection in each of the three countries. Eye-tracking measurements were obtained with a Tobii Nano system (Firmware v. 2.41.0-f2dc56e) operating at 60 Hz, using the Titta toolbox (git commit v. d6d9715) [66] and the Tobii SDK (v. 1.7.0). Visual stimuli were delivered through PsychoPy 20.2.3 on a Samsung S22E450F 22-inch display (1920 × 1080 pixels, 16:9 aspect ratio), running at a refresh rate of 60 Hz.

In Sweden, data were collected with two identical set-ups in windowless rooms while, in Japan and the US, just one setup was used in a windowless room. The participants viewed the stimuli from a distance of 65 cm. As recommended by Dunn *et al*. [67], the quality of the eye-tracker data was reported and was, across participants and eyes, given the validation in the Titta toolbox; accuracy (Sweden): 0.69 degrees (SD = 0.22); S2D-RMS precision (Sweden): 0.16 degrees (SD = 0.11); and data loss (Sweden) 2.61%; accuracy (US): 0.62 degrees (SD = 0.27); S2D-RMS precision (US): 0.18 degrees (SD = 0.14); and data loss (US) 6.72%; accuracy (Japan): 0.85 degrees (SD = 0.82); S2D-RMS precision (Japan): 0.22 degrees (SD = 0.13); and data loss (Japan) 3.44%. To restrict head movement, participants were asked to place their elbows on the desk in front of them, put their hands together and rest their chin on their hands.

Following the Titta toolbox’s standard calibration and validation procedures, participants received the instruction: *You will see a series of pictures of people. Please look at the images carefully as questions about them will follow*. Subsequently, 24 facial stimuli from the Chicago Face Database (CFD) [21]. The CFD is a free online face database. All individuals depicted in CFD images have provided their written consent to the use of their face portraits in research publications under the terms published on the CFD website. The CFD image collection protocol has been approved by the University of Chicago Institutional Review Board (protocol #H09445). Participants’ written consent is registered with the IRB. The facial stimuli were presented one at a time for five seconds each, during which participants’ eye movements were recorded. For the purpose of our study, 24 facial pictures were selected through consulting the norming data attached to the database, such as age (18–30 years), rated emotion and attractiveness and the data on a self-reported gender and race matching the subjectively rated gender and race. We considered the norming data as “ground truth” in this paper and the comparison in the analysis will always be made with the US respondents as a reference group. We excluded those faces that received average ratings in emotion and attractiveness and faces that were rated as not prototypical of the racial groups. According to the database, the norming data on racial prototypicality varied depending on rated category but for example the data was collected using the following statement: “In this survey, you will be shown pictures of Asian females. These individuals differ in terms of how much their physical features resemble those of Asian people. For example, their skin color, hair, eyes, nose, cheeks, lips, and other physical features may be more Asian (i.e., typical of Asians) or less Asian (i.e., less typical of Asians). For this study, we will show you pictures of people one at a time, and your job will be to rate how Asian-looking each person’s physical features are on a scale from ‘Less Typically Asian Looking’ (1) to ‘Very Typically Asian Looking’ (5).” Based on these data, eight pictures (four male and four female) self-identified as Asian, Black, Latino, and White were selected for the current experiment. On average, the racial prototypicality scores were 3.77, 3.71, 3.36, and 4.32, respectively. Another measurement of racial prototypicality in the norming data, provided on a scale of 0–1, showed average scores of 0.94, 0.96, 0.77, and 1, respectively [21].

The presentation order of the 24 faces was randomized for each participant. After five seconds, the image disappeared, and participants were asked to rate the skin color using the NIS skin-color scale (1–10, where 1 = dark and 10 = light). These ratings therefore reflect participants’ remembered and perceived lightness–darkness of the face rather than an exact matching of the facial skin tone to the scale. We employed the NIS Skin Color Scale because, although developed in the U.S. context, it measures perceived lightness–darkness of skin tone in a way that allows for meaningful cross-cultural comparison, capturing potential contextual differences in how the same face is perceived. Upon completing the NIS skin-color scale rating, participants were re-exposed to the facial stimuli and asked to classify each face into one of the predefined racial categories: Asian, Black, Latinx, Middle Eastern, Mixed, or White.

Although the self-identification of the faces provides four racial categories (White, Asian, Latino and Black), respondents were given two additional racial categories (Middle Eastern and Mixed) to choose from. These two racial categories are relevant not only in understanding the boundaries and social closure of racial categories across contexts but also in terms of recent discussions in the US census [68]. Upon viewing the images, respondents answered a series of questions including background questions concerning their contact with persons of different racial and ethnic backgrounds – including their experiences of international travels – and on how they understood race and ethnicity. When participants completed had the survey, they were asked to stay for an exit interview and briefing.

The whole study was piloted in Sweden first, then data were collected in Sweden (14/09/2020-18/09/2020), Japan (24/05/2022 to 30/05/2022) and the US (17/10/2022 to 26/10/2022). The questions were translated into Swedish and Japanese by a native speaker. A total of 88 college students from the Rochester Institute of Technology in the US, Keio University in Japan and Malmö University in Sweden participated in this study. To be eligible to participate, participants were required to have normal or corrected-to-normal vision (with glasses or contact lenses), no reading disabilities (self-reported) and enrolment in educational programs at a college/university level. These eligibility criteria were communicated in the information letter and selection was based on self-reporting. The students were compensated with the equivalent of a 10 US-dollar gift certificate for their participation in all three countries. In this study, we analyzed a total of 80 responses (37 from Sweden, 22 from Japan and 21 from the US) excluding those participants who self-reported as immigrants to the respective countries after the age of 13 years. (see S1 Table in the Supporting Information File for the details of the participants).

This article presents three types of data to highlight the social construction of race. First, we analyze open-ended survey responses in which participants articulate their understanding of race, offering insights into their conceptualizations and interpretive frameworks. We apply the colloquial meanings of race identified by Suyemoto et al. [7] in the U.S. context, alongside Morning and Maneri’s [10] theoretical framework on descent-based difference, as a coding strategy to explore how race is understood by respondents across the study’s three national contexts. Second, we present findings from racial ascription tasks, in which participants assign racial categories and skin color to facial images, shedding light on classification practices and perceptual tendencies. While scholars agree that visible markers are important in racial categorization, these categorizations are also flexible and context-dependent [1,69]. We therefore expect that perceptions of skin color—particularly its lightness or darkness—will vary across contexts. Finally, we present eye-tracking data that records participants’ visual attention during the ascription tasks, providing behavioral evidence of how racial cues are cognitively processed. We anticipate differences in eye movements depending on how faces are viewed, as the facial features considered salient in determining racial categories may differ by context.

We hypothesize that the social construction of race will be evident through: (1) contextual variation in how participants define race; (2) identity contestation, reflected in mismatches between self-reported racial identification and racial ascription of the faces; and (3) cross-cultural differences in eye movements during face perception. Although combining open-ended definitions of race, racial ascription tasks, and eye-tracking data enhances our analysis and offers a more holistic view of contextual differences, each method has inherent limitations. Eye-tracking does not directly capture categorization processes, open-ended and racial ascription responses may be shaped by social desirability, and ascription tasks risk reducing fluid and context-dependent identities into fixed categories. Moreover, a limitation of relying on college student samples is that their attitudes may not be fully generalizable to broader populations, as they represent a younger, more educated group whose exposure to diversity and social environments may differ from that of older adults or individuals outside academic settings.

### Data analysis

The open-ended answer to the question of how respondents understand race was coded by the first author according to the scheme suggested, based on previous studies by Suyemoto *et al*. [7] in the US and the comparative study by Morning and Maneri [10], which are mostly overlapping. The responses were coded according to the 12 coding categories suggested by Suyemoto *et al*. Coding categories were: physical characteristics, culture and ethnicity, origin and background, social group or shared experience, imposed categorization, genetic/biological, hierarchy/power/privilege, social construction, personal identity, superficial, no definitional content. We included an additional coding category – “rejection” – identified by Morning and Maneri. The answers were given in one sentence in the majority of cases (e.g., How people perceive different ethnic groups). In many cases the definition given was a combination of several codes, which means that each answer was most probably coded in multiple codes.

**Fig 1:**
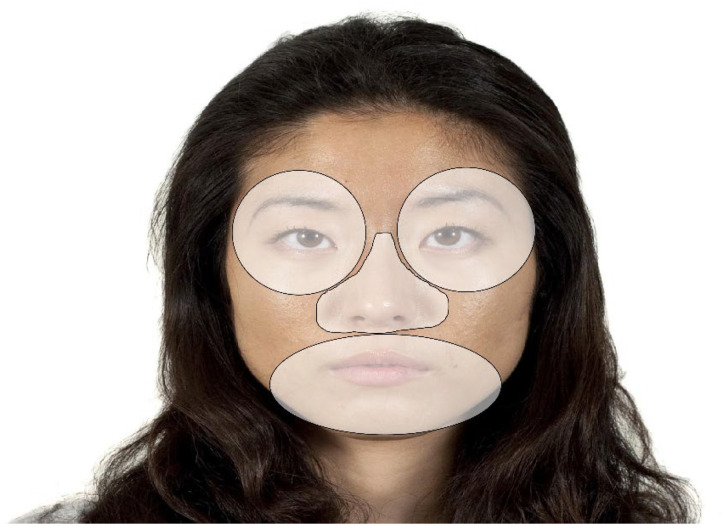
Areas of Interest (AOIs).

Reprinted from the Chicago Face Database under a CC BY license, with permission from the Chicago Face Database, original copyright 2015–2024.

Patterns of racial and skin-color ascriptions were analyzed descriptively. The eye-movement data were analyzed through total fixation time in milliseconds on areas of interest (AOIs) consisting of the eyes, mouth and nose in the face image, as illustrated in Fig 1. Fixations were detected with the I-2MC algorithm, using default settings [70].

## Results

### Contextual variation in how participants define race

Across countries, the majority of respondents defined race in terms of physical characteristics, imposed categorization and origin and background, as summarized in Table 1 below (see S1 Table and S2 Table for examples of definition). However, we observe differences between countries – for example, race is defined largely as culture and ethnicity and as hierarchy, power and privilege by Swedish respondents. Many Japanese respondents did not give any definitional content to the question. US respondents defined race mainly as physical characteristics; however, there were no respondents. Our sample across countries also shows that very few rejected the concept of race itself, contrary to the findings by Morning and Maneri [10].

**Table 1:** Three-country comparison of how race is defined.

	Sweden (N = 37)	Japan (N = 22)	US (N = 21)
Physical characteristics	7	8	12
Imposed categorization	3	3	5
Origin and background	5	3	4
Culture and ethnicity	6	0	3
Ancestry, heritage history	1	0	2
No definitional content	1	9	2
Hierarchy, power and privilege	7	3	1
Genetic, biological	0	0	1
Rejection	1	2	1
Social group or shared experience	1	1	0
Social construct	0	0	0
Personal identity	0	3	0
Superficial	0	0	0

### Identity contestations and perception of skin color

Comparing the norming data indicating the self-identified race of face and the racial identifications made by the survey respondents for each face, we look at the potential identity contestation between the contexts. Table 2 shows the percentage of racial ascription and self-reported race-matching. We see that the identity contestation for Asian and White faces is small (values close to 100%) across the three countries. On the other hand, there were higher identity contestations for Black faces and, especially, for Latino faces. Black faces were the most often ascribed as mixed across context (13.9%, 20.6% and 10% in Japan, Sweden and the US respectively) and heavily as Latino in Japan (22.5%). Latino faces were often ascribed as Middle Eastern (23%, 42.1% and 25.5%) or mixed (17.8%, 16.3% and 24.5%) across the three contexts.

**Table 2:** Percentage of matching racial ascription in relation to the self-reported racial identification in the facial database.

	% Asian	% Black	% Latinx	% White
Japan	87	55	33	96
Sweden	93	78	47	97
US	92	81	52	93

The mean NIS skin-color identification (1–10) for faces according to the self-identified race shows that Japanese respondents give slightly higher skin-color shades for faces self-identified as Asian and Black compared to our US respondents, while Swedish respondents give slightly lower skin-color shades for Black and Latinx faces.

**Fig 2:**
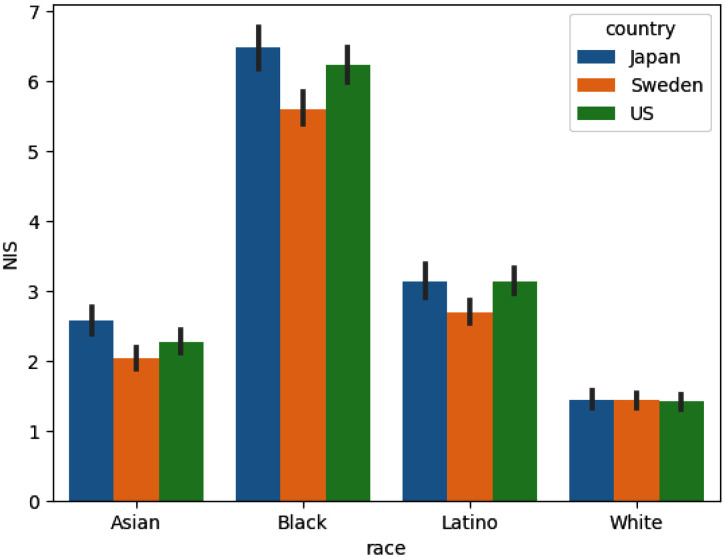
Three-country comparison of mean NIS skin-color ratings per self-identified race of the face. Note: A low NIS value indicates that the skin color is perceived as bright, whereas a high NIS value indicates that the skin color is perceived as dark. Error bars represent 95% confidence intervals.

Fig 2 shows how NIS is related to the self-identified race of the faces and the country of origin of the participants. To test whether there were any statistical differences in NIS between faces with different racial backgrounds, we ran a separate ANOVA for each racial background. There were significant main effects for Asian (*F*(3, 22) = 9.95, p < 0.01), Black (Black: *F(*3, 22) = 12.22, p < 0.01), and Latino faces (Black: *F*(3, 22) = 7.07, p < 0.01). Tukey post-hoc pairwise comparisons revealed significant differences (all p < 0.01) between Japan and Sweden for Asian faces (p < 0.01), between Sweden and Japan and Sweden and the US for Black faces and Sweden and Japan and Sweden and the US for Latino faces.

**Fig 3:**
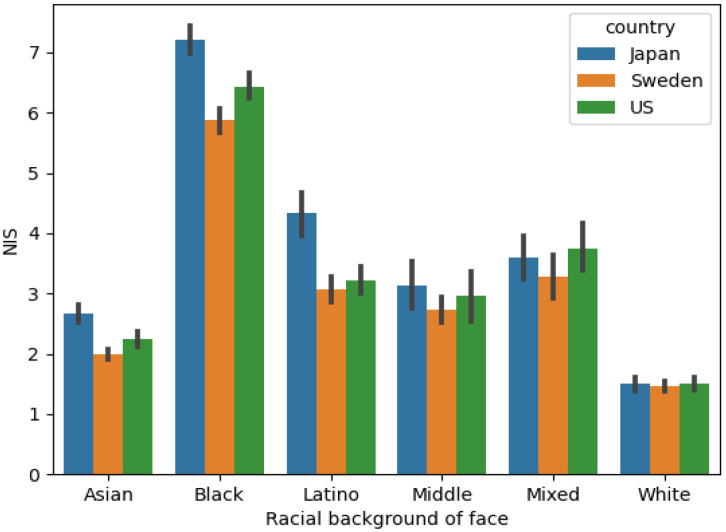
Three-country comparison of NIS skin-color ratings (right) per ascribed race of the face.

The above analysis of NIS skin-color ratings per self-identified race showed that Swedish respondents assigned lighter skin color to all groups except for faces self-identified as White; a similar pattern can be observed again in the analysis of the reported NIS skin color according to the racial ascription made by the respondents between contexts. Compared to the US respondents, Swedish respondents assign in general lighter skin-color to all faces except for those ascribed as White. Fig 3 shows how NIS is related to the assigned racial group of the faces across countries.

There were significant main effects for Asian (F(3, 22) = 12.42, p = 0.00), Black (F(3, 22) = 17.84, p = 0.00) and Latino (F(3, 22) = 19.42, p = 0.00) faces. Tukey post-hoc pairwise comparisons revealed significant differences (all p < 0.01) between Japan and Sweden and Japan and the US for Asian faces (p < 0.01), between all countries for Black faces and Sweden and Japan and Sweden and the US for Latino faces.

### Cross-country differences in eye movements

Reprinted from the Chicago Face Database under a CC BY license, with permission from the Chicago Face Database, original copyright 2015–2024.

**Fig 4:**
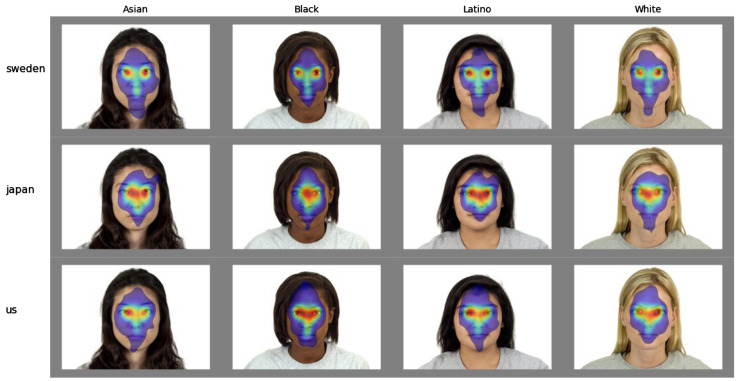
Heatmap of total fixation time across self-identified race.

**Fig 5:**
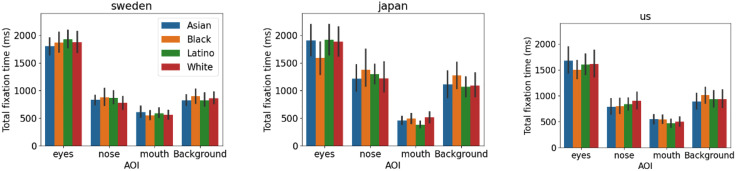
Average total fixation time on different parts of the face: areas of interest or AOIs for faces from different racial backgrounds. Note: Error bars span 95% CI; ‘ws’ denotes ‘whitespace’, i.e., any area in the image that does not belong to the eyes, mouth or nose (cf. the ‘Measures and data analysis’ section for more detail).

The results from the three contexts confirm those of previous studies which showed that people attend to eye regions much more than other principal facial features such as the nose and mouth [50] (see Fig 4 Heatmap). The total fixation time on different parts of the face is shown in Fig 5, separately for the different self-reported racial backgrounds of the faces.

We conducted three separate one-way ANOVA, which showed no statistically significant differences in total fixation time between faces of different racial backgrounds, for any of the face regions (Table 3).

**Table 3:** Total fixation time on faces of different racial backgrounds per face region.

Country	AOI (face region)	Statistics
Sweden	Eyes	F(3, 40) = 0.35, p = 0.79
	Mouth	F(3, 38) = 0.35, p = 0.79
	Nose	F(3, 39) = 0.53, p = 0.66
	Background	F(3, 39) = 0.36, p = 0.78
Japan	Eyes	F(3, 24) = 1.16, p = 0.33
	Mouth	F(3, 23) = 1.87, p = 0.14
	Nose	F(3, 23) = 0.29, p = 0.83
	Background	F(3, 24) = 0.66, p = 0.58
US	Eyes	F(3, 24) = 0.37, p = 0.77
	Mouth	F(3, 22) = 0.76, p = 0.52
	Nose	F(3, 24) = 0.50, p = 0.69
	Background	F(3, 24) = 0.41, p = 0.75

However, for faces of some racial backgrounds, there were significant main effects in how much participants in different countries looked at the eyes, mouth, background and, in particular, the nose (see Table 4).

**Table 4:** Total fixation time on face regions per respondents of different countries.

Race	AOI (face region)	Statistics
Asian	Background	F(3, 351) = 3.34, **p = 0.04**
Black		F(3, 351) = 5.03, **p < 0.01**
Latino		F(3, 351) = 2.73, p = 0.07
White		F(3, 351) = 2.09, p = 0.13
Asian	Eyes	F(3, 352) = 0.88, p = 0.42
Black		F(3, 352) = 3.38, **p = 0.04**
Latino		F(3, 352) = 2.75, p = 0.07
White		F(3, 352) = 1.49, p = 0.23
Asian	Mouth	F(3, 329) = 2.23, p = 0.11
Black		F(3, 329) = 0.47, p = 0.63
Latino		F(3, 329) = 6.62**, p < 0.01**
White		F(3, 329) = 0.61, p = 0.54
Asian	Nose	F(3, 347) = 8.20**, p < 0.01**
Black		F(3, 347) = 7.07, **p < 0.01**
Latino		F(3, 347) = 11.13, **p < 0.01**
White		F(3, 347) = 5.98, **p < 0.01**

## Discussion

This study aims to empirically investigate the social construction of race by examining how the concept of race is understood and how racial categories are flexibly applied by college students from three distinct national contexts: Japan, Sweden, and the United States. While few empirical studies have examined how racial appraisal differs from self-identification [23], this study addresses that gap by providing a comparative and contextual analysis of how race is perceived, categorized, and cognitively processed across these settings. Drawing on three types of data—open-ended definitions of race, racial and skin-color ascription tasks, and eye-tracking measures—the study highlights contextual differences in how respondents perceive race, shaped by their respective sociocultural environments. The findings contribute to a deeper understanding of how race is conceptualized, enacted, and cognitively processed, offering new insights into the comparative dynamics of racial categorization and providing empirical evidence that strengthens our theoretical understanding of race as a socially constructed phenomenon.

Across contexts, the majority of respondents defined race in terms of physical characteristics, imposed categorization and origin and background. These patterns show a colloquial understanding about the social reality of race across contexts – that race deploys physical characteristics to create structures of hierarchy. However, we also observe some differences between the three countries. The subtle differences in how respondents answered the question, especially in providing non-definitional answers or defining them in terms of culture and ethnicity, do indicate the contextual differences in understanding the word race. Swedish respondents defined race largely as culture and ethnicity and as hierarchy, power and privilege. Defining race as culture and ethnicity reflects, on the one hand, the avoidance of using the word ‘race’ in Sweden, where the concept of race is often replaced with cultural or ethnic terminology. On the other hand, there are political awareness and numerous reports about the existence of structural discrimination and the inequity that exists between those who are defined as having a Swedish background (born in Sweden having two Swedish-born parents) and those with an immigrant background (foreign-born or born in Sweden with two foreign-born parents), thereby demonstrating a conceptual understanding how race functions [17,71]. In Japan, the concept of race is not commonly addressed; instead, the focus is on the idea and identity of the Japanese being a specific race [72,73] and this might be reflected in the fact that respondents could not give any definitional content when asked what race means to them. As in earlier studies, US respondents for our survey also defined race as physical characteristics [23]; however there were no respondents who defined race in terms of social group and shared experience [7].

Scholars agree that visible markers – often considered important for racial categorizations – are flexible and context-dependent [1,33]. Feliciano [23] has shown that in the U.S., racial classification of faces varies depending on the respondent’s own race and gender. Our findings similarly demonstrate that the same face can be racially ascribed differently and perceived as having lighter or darker skin tones, despite a shared colloquial understanding of what race means. These results underscore the contextual nature of racial perception and point to identity contestation across national settings.

In the U.S. sample, the inclusion of MENA and Mixed categories may have introduced greater flexibility in the racial ascription of faces self-identified as Black and Latinx. In contrast, identity contestation was not observed for faces self-identified as Asian or White across the three countries. Notable differences emerged in the racial ascription of MENA in Sweden and Latinx in Japan. These findings align with demographic realities: MENA populations (particularly Syrians and Iraqis) represent a salient minority in Sweden, comprising approximately 8% of the population, while Brazilians are among the largest ethnic minority groups in Japan.

Given that racial categories are socially constructed, the observed variation in identity contestation is not unexpected. However, it is significant in empirically illustrating how racial categorization and ascription operate differently across contexts. The Latinx category, in particular, is highly contested and flexible, encompassing diverse populations across national boundaries [74–76]. Even within the U.S., the Latino category is fluid, shaped by racial and socioeconomic factors, often resulting in mismatches between self-identification and external classification [77–79].

The variation in the flexibility of the Black category is also noteworthy. In the U.S., racial ascription of Blackness has historically been shaped by the “one-drop rule,” which classifies individuals with any Black ancestry as Black [16,38]. This contributes to the relative stability of the category in the U.S., but such frameworks may not apply in Sweden or Japan. While global movements like Black Lives Matter have contributed to the transnational circulation of racial ideas, the identification and ascription of Blackness remain contextually distinct [75,80]. The growing recognition of Afro-Latinx identities further complicates these dynamics [81].

What merits further theorization is the relative stability of the Asian and White categories. Scholars have argued that the globalization of Whiteness shapes racial perceptions across contexts [82], and racial triangulation theory [83,84] offers a framework for understanding how Asian and White identities are positioned in racial hierarchies. In Japan, the consistent ascription of Whiteness aligns with contemporary notions of racial privilege and national identity, where “Whiteness” and “Japaneseness” are intertwined [20,85,86].

Our findings also reveal contextual differences in skin-color perception, except for faces both self-identified and ascribed as White. While the same skin tone may be perceived differently across contexts, the patterns of variation are significant. Swedish respondents generally perceived lighter skin tones across all non-White groups, while Japanese respondents tended to perceive darker skin tones, particularly for faces self-identified as Latino and Asian. These patterns were consistent with racial ascription: Japanese respondents reported significantly darker skin tones for faces ascribed as Black, Asian, and Latinx, while Swedish respondents reported lighter tones overall. Statistically significant differences were observed for faces ascribed as Asian and Black, but not for those ascribed as White.

These results underscore the importance of context in shaping racial perception. Scholars have long emphasized the role of Whiteness in the Swedish and Nordic contexts [87]. Our findings—though based on a non-representative sample—offer one of the first empirical indications of the role of phenotype in identifying racial Whiteness in Sweden. Similarly, the darker skin-color perceptions among Japanese respondents may reflect historically rooted aesthetic norms, such as the desirability of *shiroi* (white) skin and the negative connotations of *kuroi* (black) skin [88].

We expected that participants’ eye movements would also vary across contexts, as the facial features considered important for determining racial categories may differ depending on sociocultural norms. However, we did not observe any differences in visual attention paid to eyes, nose and mouth between faces of different racial backgrounds. This is contrary to previous studies which show, for example, Western participants focused more on the eyes of White than Asian faces and Eastern participants focused more on the eyes of Asian than White faces [54,89]. However, we did find that the time spent looking at the eyes showed statistically significant differences across the three contexts for all racial groups. Previous studies have shown distinct patterns of eye movements for people raised in different geographical locations and/or in different cultures – a finding which our study confirms. In particular, our study confirms the results from face-recognition tasks which show that White participants from a Western culture fixated more on the eye regions and mouths of both White and Asian targets, while Asian participants from an Eastern culture focused more on the nose and center of the face [58,90,91]. The differences in eye movements which we observe in the current study indicate the cultural differences in processing visual information [92], rather than cross-race effects or which facial features are considered important in determining racial groups. The eye-tracking data, which do not show major cross-national differences, underscore the importance of context in shaping racial perception. As mentioned earlier, the majority of respondents define race in terms of physical characteristics, imposed categorization, and origin or background—indicating that physical features are used to construct hierarchical structures. The finding that there are no differences in visual attention to the eyes, nose, or mouth between faces of different racial backgrounds across contexts further strengthens the argument that it is not the objective physical characteristics per se that matter, but rather how these characteristics are used in racial categorization. The ways in which they are perceived and deployed for racial ascription are flexible and context-dependent. In other words, while basic perceptual processes involved in scanning faces appear relatively stable across populations, the meanings attached to those same features—and their role in racial classification—are far more sensitive to social, cultural, and historical context. While our results show no observable differences in visual attention patterns across racialized faces, the study design does not allow us to determine whether these similarities reflect underlying perceptual mechanisms or other factors.

## Conclusion

The empirical results presented above highlight the contextual flexibility of racial categorization and provide compelling evidence for the social construction of race. While these findings are significant, they must be interpreted with caution due to certain limitations. The experimental setup varied slightly across the three countries—for example, in room settings and screen configurations—which may have influenced participants’ responses. Moreover, the small sample was limited to college students in Malmö, Rochester, and Tokyo, which constrains the generalizability of the findings, particularly with regard to national contexts. Although the overall sample size for this study (N = 80) is well within the range typically used in lab-based eye-tracking research [93,94], the country-specific subsamples are relatively small (Japan, n = 22; U.S., n = 21). This limits the precision of cross-national comparisons and warrants caution when interpreting country-by-condition effects. At the same time, the primary analyses draw on trial-level visual attention data, including multiple fixations per stimulus and predefined areas of interest (AOIs), which provide high-resolution behavioral measurements and substantial within-person information even when between-country sample sizes are modest. Following best practices, we therefore interpret cross-national patterns as suggestive rather than definitive and emphasize convergence across attention-allocation metrics (e.g., dwell time) and downstream judgments. Future research with larger, quota-matched samples will be necessary to validate and extend these findings.

Despite these limitations, the study offers valuable empirical insights into how race is perceived and enacted across different sociocultural contexts. The findings raise important questions about salience and ingroup biases [95,96], as well as the global dynamics of race and Whiteness [97,98]. While the categories “Asian” and “White” appear relatively stable across contexts, the ascription of “Black,” “Latinx,” “MENA,” and “Mixed” identities is more fluid and context-dependent—shaped by the local salience of these groups. Moreover, beyond the local salience of specific racialized groups and differences in minority population size across contexts, context-dependent norms and national discourses about race—as well as the avoidance of talking about race—may also shape how racial groups are perceived and categorized.

Although scholars have long called for comparative approaches to the study of race, we still know relatively little about how racial categories and practices of classification vary across national and cultural boundaries. This article represents a first step toward addressing that gap by empirically examining the social construction of race in three distinct contexts. The results showing the different patterns of racial ascription to the same faces indicate that racial categorization and racialized identity may vary across cultural contexts. Being racially ascribed and categorized differently across countries may have implications for individual experiences of identity and inclusion, particularly in the context of migration. We call for further research that systematically explores how understandings of race, racial identity, and racial perception converge and diverge across different socio-historical settings, and how these differences may shape individual racial identity and experiences of inclusion.

Future research should extend this study to broader and more diverse demographic groups and larger samples to better understand how perceptions of race and processes of racial categorization may differ across social and cultural contexts. Expanding the study population beyond college students would strengthen the external validity of the findings and provide a firmer empirical basis for understanding how perceptions of race and practices of racial ascription vary across different social and cultural contexts.

Our findings indicate a contribution to understanding the intersection between visual perception and social cognition of race, where one looks and how one categorizes what one sees, as the patterns we observe suggest that participants do not rely solely on physical characteristics when classifying racial groups. Rather, their interpretations of these characteristics appear to be shaped by socially and culturally defined contexts. Given the design and scope of the present study, we cannot assess the perceptual processes involved in scanning faces or how meanings are attached to physical characteristics and used in racial classification. Understanding these processes is essential for determining how participants’ racial categorizations arise from cultural and social learning and for clarifying how racial perception is context dependent. We therefore note this as a limitation and suggest that future research should address this distinction more directly.

## Supporting information

S1 Table Overview of respondents. (DOCX)

S2 Table Examples of answers given to the question “What does race mean to you?”. (DOCX)
