# Supplementary material for: What is socially constructed about race? A cross-national study of racial perception and categorization
Source: PLoS One. 2026 Aug 12;21(8):e0351831. doi: 10.1371/journal.pone.0351831 (PMC13465800; doi:10.1371/journal.pone.0351831)
Supplement: Supplementary file 1 [file pone.0351831.s001.docx]

**Appendix**

**Table A1. Overview of respondents**

|  | **Sweden (N=37)** | **Japan (N=22)** | **US (N=21)** |
| --- | --- | --- | --- |
| **Age** | 25.46 (18–50) | 21.1 (20–23) | 20.3 (18–28) |
| **Gender** |  |  |  |
| Male | 8 | 17 | 10 |
| Female | 28 | 5 | 10 |
| Prefer not to answer | 1 | 0 | 1 |
| **Self-reported race** |  |  |  |
| Asian | 0 | 22 | 7 |
| White | 24 | 0 | 9 |
| Middle Eastern | 4 | 0 | 0 |
| Black | 1 | 0 | 3 |
| Mixed | 8 | 0 | 2 |
| **Native-born** | 34 | 21 | 19 |
| **Educational program** |  |  |  |
| Social science | 32 | 22 | 20 |
| Science | 5 | 1 | 1 |
