# Supplementary material for: What is socially constructed about race? A cross-national study of racial perception and categorization
Source: PLoS One. 2026 Aug 12;21(8):e0351831. doi: 10.1371/journal.pone.0351831 (PMC13465800; doi:10.1371/journal.pone.0351831)
Supplement: Supplementary file 2 [file pone.0351831.s002.docx]

**Table A2. Examples of answers given to the question “What does race mean to you?”**

|  | **Sweden** | **Japan** | **US** |
| --- | --- | --- | --- |
| **Physical characteristics** | For me, it is mainly connected to when the color of your skin deviates from what is considered Swedish and classic Swedish features | I feel that it is about the color of the skin | To me, race means the color of your skin |
| **Culture and ethnicity** | I see it as the same as ethnicity |  | Similar to ethnicity |
| **Origin and background** | It means that you divide people into different caegories based on origin | Regional roots | Race is a textbook classification of where you are from geographically and the characteristics pertaining to that |
| **Social group or shared experiences** |  | A word that gives the impression that there are more people with a sense of belonging even more than ethnicity |  |
| **Ancestry, heritage, history** | Provision of various factors that identify a person's background and/or ancestry |  | Race to me is my ethnicity and where my history begins from past generations |
| **Imposed categorization** | To categorize people from different countries into groups | Something that makes you aware of the differences | The generalized group of people wirth whom you have the smallest common denominator |
| **Genetic, biological** |  |  | Race is your genetics, your skin color or how someone looks on the outside |
| **Hierarchy, power and privilege** | To exotify or discriminate against someone because of their ethnicity | When you hear the word ‘race’, it is a word that is associated with racism | Race is the the type of people that one is derived from through generations. It affects the way one looks and how one might be treated in certain other cultures |
| **Personal identity** |  | Factors that continue to bind people |  |
| **No definitional content** | Segregation | Feel the international scale | Not much. If you are a good person, you are a good person |
| **Rejection** | Race of humans is a strange concept | I have a negative image of it | Means nothing, its a made-up word that divides people |
